# The Application of the Principles of Responsible AI on Social Media Marketing for Digital Health

**DOI:** 10.1007/s10796-021-10191-z

**Published:** 2021-09-13

**Authors:** Rui Liu, Suraksha Gupta, Parth Patel

**Affiliations:** 1 Newcastle University Business School, Newcastle University, 5 Barrack Road, Newcastle upon Tyne, NE14SE Tyne and Wear UK; 2 Discipline of Management & Human Resources, Australian Institute of Business, 1 King William Street, Adelaide, 5000 South Australia Australia

**Keywords:** Responsible AI, Social media marketing, Digital health, Information sharing theory, Consumer trust theory, Technology acceptance model

## Abstract

Social media enables medical professionals and authorities to share, disseminate, monitor, and manage health-related information digitally through online communities such as Twitter and Facebook. Simultaneously, artificial intelligence (AI) powered social media offers digital capabilities for organizations to select, screen, detect and predict problems with possible solutions through digital health data. Both the patients and healthcare professionals have benefited from such improvements. However, arising ethical concerns related to the use of AI raised by stakeholders need scrutiny which could help organizations obtain trust, minimize privacy invasion, and eventually facilitate the responsible success of AI-enabled social media operations. This paper examines the impact of responsible AI on businesses using insights from analysis of 25 in-depth interviews of health care professionals. The exploratory analysis conducted revealed that abiding by the responsible AI principles can allow healthcare businesses to better take advantage of the improved effectiveness of their social media marketing initiatives with their users. The analysis is further used to offer research propositions and conclusions, and the contributions and limitations of the study have been discussed.

## Introduction

The Internet has emerged as an imperative tool for people seeking health knowledge from physicians, public health workers, and laypeople (Brownstein et al., 2009). Users of social media particularly spend much of their daily time on online applications where people discuss their wellbeing, broadcast thoughts, experience and feelings about their health, and generate many relevant data. Blogs, Facebook groups and Twitter have all been identified as resources for patients to communicate with each other. For example, Twitter users can mark their tweets as public to allow other users to read and retweet to others. According to two web-based patient registries, 83 % of patients used social media to find out information about their diagnosis or test results, 73 % of them read posts from rare disease groups or organizations, 67 % of them engaged in conversations about their diagnosis and 58 % of them used social media to connect with others to seek help (Rocha et al., 2018). Social media platforms offer public health authorities and researchers the chance to collect health-related information generated by patients. In emergency and catastrophe management, social media has also become a significant communication route (Mirbabaie et al., 2020). Along with AI and machine learning, social media has been used in the prediction, detection, and treatment solutions for mental health care via web and smartphone applications (D’Alfonso, 2020). Accountable technology, therefore, is consistently changing and co-shape human actions as it offers answers to the ethical questions of how people can act (Peters et al., 2020).

Researchers in the computing and information systems fields have done several pioneer practical explorations on harnessing AI and machine learning to make digital inventions in improving user experience and optimize personalized mental health care, as well as a therapeutic intervention. For example, to relieve the cost of long waits for clinic visits, using AI instead of costly clinical staff, and to keep the “worried well” from heading to the hospital, an artificially intelligent chatbot on a mobile app was created to give British people diagnostic advice on common ailments without human interaction (Olson, 2018). It was also a good replacement for the health advice telephone line which couldn’t show how many patients take the advice, and AI bots could track that data. Natural language processing (NLP) is one of the AI-powered tools in analyzing the language of humans and solutions to mental health. Machine learning and NLP technologies have explored the potentials for extracting useful health information from substantial data of the Internet and have earned substantial achievements (Dredze, 2012). User-generated content comes from a personal digital device and social media interactions, and the individual’s ‘digital exhaust’ is continuously creating a growing reservoir of data for NLP studies and can be mined for behavioural and mental health insights, deep and real-time data analysis potential cultivation (D’Alfonso, 2020). The widespread use of social media combined with AI, help people with behaviour and mental illness to be effectively treated at a relatively low cost by filling in the gap between individuals’ demands for healthcare resources and those with abundant scientific and efficient access. However, social media users may perceive risks and uncertainties when sharing their health data on social media to achieve better health. The level of user technology acceptance is determined by some factors including perceived usefulness, perceived ease of use, behavioural intention, and other contextual factors.

### Research Gaps

In terms of research gaps, this research is important for three reasons. Firstly, even though how to manage AI responsibly has invoked some debates in broad scope, a systematic discussion about responsible AI principles is limited. In a study for examining ethical AI principles in maintaining their deployment in organizations, Eitel-Porter (2021) focuses solely on the trust, fairness, and privacy ingredients of AI, and does not include data security. Arrieta et al. (2020) established that fairness, accountability, and privacy should be regarded when bringing AI models into practice. Under increasing use of AI in high-stakes decision-making, another research on the design of a contestation process has found its effects on the perception of fairness and satisfaction; it also found that a lack of transparency can be due to design challenges. (Lyons et al., 2021) Lima & Cha (2020) discussed three notions of responsible AI, including blameworthiness, accountability, and liability for actions of AI. Ghallab (2019) identified three important risks in AI deployment (i.e., the safety of AI applications, the security, and privacy for individual users, and the social risks). Consequently, the current research and appliance in the field were restricted to a few separate elements of responsible AI principles without a comprehensive understanding. Secondly, despite the widespread use of social media in promoting, the application of responsible AI in facilitating digital health through social media is scarce; thirdly, associated practical investigations of responsible AI are absent and therefore the research lacks an empirical foundation. This paper has endeavored to fill in the gaps by proposing a set of responsible AI principles and putting eight principles into digital health practices that are critical in determining the effectiveness of responsible AI.

### Theoretical Issues and Limitations

While many cross-disciplinary experts have acknowledged the concerns of blameworthy irresponsible AI at a fast rate and published many AI principle models, the attempts of these principles to translate into AI practices is still in their infancy (Benjamins, 2020; Cheng et al., 2021; Scantamburlo et al., 2020). The reason at its core relies on their abstract nature that makes practitioners feel difficult to operationalize (Scantamburlo et al., 2020), and companies might overlook the financial rewards of responsible AI and treat it as the sole way to avoid risks (Cheng et al., 2021). Also, there is no consensus about which principles should be included for responsible AI and how to practically deal with these issues in organizations (Gupta et al., 2020; Benjamins, 2020).

The majority of the attention has mainly focused on the unintended negative impacts of responsible AI on ethical issues rather than intended and controllable impacts because the intended motivation of developers seems less likely to release a new technology blindly (Peters et al., 2020; Wearn et al., 2019). Relatively little attention has been paid to understand proactive organizational ethical efforts. Without a detailed understanding of specific AI tools from a third party, an organization may trigger biases that negatively impact the brand and creates compliance issues. For example, Eitel-Porter (2021) clarified three categories of responsible AI: compliance and governance, brand damage and third-party transparency. Specifically, biases in training data may lead to recruitment apps to favour one gender which breaches anti-discrimination laws; AI algorithms may prioritize the delivery services in affluent areas, which breaches social norms and taboos.

### Managerial Research Issues

Despite the opportunities and benefits of AI, experts revealed various risks regarding the interests of the organizations and their stakeholders (Clarke, 2019). The concept of responsible AI has been stated which seeks to support the design, implementation, and use of ethical, transparent, and accountable AI solutions, aiming at reducing biases, facilitating fairness, equality, interpretability, and explainability (Trocin et al., 2021). The hope for the development of a Good AI Society has been proposed accordingly (Floridi et al., 2018). They acknowledged the impact of AI technology for promoting human dignity and human flourishing, and also identified sequent problems of privacy issues, unfairness, unreliability, and unsafety, maleficence which emanated as detrimental barriers for the sustainability of organizations. For instance, mental health care has raised particular ethical and legal considerations as well as the need for regulation by its nature, such as privacy violation and rooted prejudice (D’Alfonso, 2020). Ethics have an impact on people’s digital health in terms of privacy, fairness, inclusiveness, transparency, accountability, privacy, security, reliability, and safety (ibid.). The expectation and the response of users are changing. As a result, the design, assessment, and use in diverse industries of ethical AI should be ongoing and iterative. Floridi et al. (2018) proposed an ethical framework for a Good AI Society. They constructed this framework from the researchers’ point of view, hoping the framework can be undertaken by policymakers and each stakeholder. The managerial research issues in this research are to examine the solutions to ethical problems faced by AI and its use in social media. This research seeks to bridge the responsible AI principles and theories to business practices particularly in the healthcare industry.

### Research Questions, Aims, and Objectives

In light of the abovementioned gaps, this paper attempts to answer the following research questions:

- RQ1) How organizational appliance of responsible AI principles in practice can contribute to the digital health maintenance of social media consumers? And
- RQ2) How does consumer trust and data accuracy moderate the relationship between responsible AI principles and activities?

To be specific, the aims of objectives can be clarified as follows:

1. To construct a set of responsible AI principles which can guide organizational responsible AI activities.
2. To address the ethical dilemma of responsible AI solutions in social media.
3. To provide reliable evidence to guide professionals in improving the delivery of digital health.

### Theoretical and Practical Contributions

Thus, this research constructs and proposes a conceptual model that attempts to elaborate on the effective responsible AI activities from both the organizational level and consumer level, by surveying the responses of healthcare managers and employees towards their expectations, feelings, experiences, and concerns to validate the model. The authors of this research have incorporated three typical frameworks of responsible AI principles practically from a company (Microsoft AI, 2020) and theoretically from two articles (Clarke, 2019; Floridi et al., 2018) into a discussion, to examine whether the existing responsible efforts made by AI designers, operators and other users in the healthcare industry are liable or not. Further from the impacts of responsible AI activities on social media marketing efforts and influences of these efforts on digital health, this new developed conceptual model would also help better understand the moderating role of consumer trust and data quality in the influencing mechanism, because most of the existing research on consumer trust is focusing on the trust of online information, while this paper would switch the focus on whether consumer trust of the platforms and technology might take effects on social media marketing of digital health in organizational level. Additionally, relevant research on the theory of information sharing use in inspecting social media performance is so scarce and requires further consideration. To seek healthcare expertise views, industrial professionals, policymakers, and others can take actions and make changes, set up policies, regulations, and laws more responsibly in the public information sharing by stakeholders such as patients and clinicians. Consumers would feel more secure about sharing their health data on social media since it is no longer risky when they leave a footprint on social media, to achieve better health more cheaply and conveniently. This paper also showcases the opportunities and challenges under the responsible AI paradigm.

## Literature Review

### What is Responsible AI?

Responsible AI has been used interchangeably with ethical AI or the responsible use of AI. Eitel-Porter (2021) defined the term as the practice of using AI with good intention, to empower employees and businesses and create a fair environment for customers and society, ultimately enabling organizations to generate trust and bring AI deployments to scale. Taylor et al. (2018) regarded responsible AI as an umbrella term, which investigates legal, ethical, and moral viewpoints of autonomous algorithms or applications of AI that may be crucial to safety or may impact people’s lives in a disruptive way. AI incorporates a broad range of techniques and approaches of computer science to simulate human intelligence in machines that are programmed for thinking like humans and mimic human behaviours, capable of performing tasks. During the past 80 years after World War II, the advances of AI stimulate the invention, innovation, and investment in computing skills (Clarke, 2019). Some AI technologies are relatively longstanding, such as online robots for counselling, while others are more recent for example computational modelling used by social media companies to identify users at risk of self-harm. The identification of AI technology and associated appliances in different situations with the changes of markets and society engendered both opportunities and threats to organizations.

AI technologies may be underused, overused or misused, associated with fear, ignorance, misplaced concerns, or excessive reaction (Floridi et al., 2018; Meske et al., 2020). According to Eitel-Porter (2021), unintended negative consequences for organizations may occur when AI applications have been done without care. For example, faulty or biased AI applications which breach the risk compliance and governance may damage the brand. The use of AI, therefore, should be coupled with good innovation and positive execution of this technology abiding by moral principles and social norms. The larger number of people who enjoy the opportunities and benefits of AI, the more responsibilities are essential in terms of what type of AI to develop, how to use it, and who can use it properly. There is a great need to guide how AI should be permitted, how it can be used responsibly, and when it should be discouraged or forbidden raises without precedents (Gooding, 2019). Many common pitfalls raise risks for an organization: rushed development, a lack of technical understanding, improper quality assurance, use of AI outside the original context, improper blends of data, and reluctance by employees to raise concerns (Eitel-Porter, 2021). Consequently, proper use and the proposal of accountable guidelines of AI have emerged as crucial concerns for the public and the organizations.

There are a number of factors that impact the behaviour of AI systems, so the term ‘responsible AI’ has been used in different fields, aiming at mitigating bias in data collection and design of the algorithm and engendering better interpretability and explainability (Contractor et al., 2020). Nevertheless, although the significance of responsible uses of AI has been realized and discussed in the past few years and concerning diverse responsible AI principles and frameworks, the research on responsible AI remains at the theoretical stage, and it seems to be difficult to apply it to the practical hierarchy or to apply it to industries broadly. Another problem with responsible AI research is that most responsible AI guidelines are useful and can help shape policy but are not easily enforceable. Arrieta et al. (2020) presented a theoretically oriented analysis for concepts, taxonomies, opportunities, and challenges towards responsible AI. Contractor et al. (2020) have built a framework and offered suggestions to relieve the concerns about inappropriate or irresponsible use of AI through licensing on software and data to legislate AI usage. While Eitel-Porter (2021) asserted that responsible AI needs mandated governance controls involving methods for managing processes and enforcing audit trials. All of these articles solely concentrate on the abstract conceptual level of discussions such as guidance and principles.

In the AI-enabled social media marketing field, an overload of knowledge, false reports, lack of signal specificity, and sensitivity to external forces such as the interest of the media might greatly limit the realization of the capacity of health care for public health practices and clinical decision-making (Brownstein et al., 2009). The researchers also point out the user privacy issues (Dredze, 2012; Kapoor et al., 2018). Users of social media hold privacy and ethical issue concerns and expectations towards AI, such as algorithms that infer unstated user demographics or diagnoses from public data (Dredze, 2012). Responsible AI should tackle the tension between incorporating the benefits and mitigating the potential harms of AI and avoiding the misuse and underuse of AI (Floridi et al., 2018). Integrating ethics into AI allows organizations to take advantage of the social norms, values, and culture, participate in those socially acceptable or preferable, legally unquestionable activities, and prevent and minimize costly mistakes (ibid.). Hence, examining the responsible use of AI in social media emerges as an essential activity for AI-associated organizations and researchers.

Responsible AI is about answering who determines which are the alternatives and how to implement for ethical decision-making by AI systems, consistent with societal, ethical, and legal requirements. Existing approaches to the implementation of ethical reasoning can be divided into three main categories: top-down approaches, bottom-up approaches, and hybrid approaches. Top-down approaches are to implement a given ethical theory and apply it to a particular case; bottom-up approaches are to aggregate sufficient observations of similar situations into a decision and infer general rule from these independent cases; hybrid approaches combine the benefits of bottom-up and top-down approaches in support of a careful moral reflection (Singer, 2020). From a deontological view, this paper leverages the top-down approaches by applying the current responsible AI frameworks to the social media health cases and then describes what people should do in this specific situation, judge the ethical aspects or ‘goodness’ of behaviours in AI systems and to ensure ethical acceptance and social acceptance for the ethical reasoning by AI systems.

### The AI Ethical Principles

There are many researchers and business organizations that have produced different categories of responsible AI principles, approaches, and practices throughout the existing AI practice and literature (Arrieta et al. 2020; Benjamins et al. 2019; Clarke, 2019; Ghallab, 2019; Lima & Cha, 2020; Lyons et al. 2021). For example, Arrieta et al. (2020) stated that fairness, ethics, transparency, security and safety, accountability and privacy should be considered as AI models that need to be implemented in the real world and this would bring a gradual process in increasing corporate awareness around AI principles. According to the European Commission’s ‘Ethics Guidelines for Trustworthy AI,’ trustworthy AI should be lawful, ethical, and robust both from a technical perspective and social environment (European Group on Ethics in Science and New Technologies, 2018). Beijing Principle, which is the first principle of artificial intelligence for children, has been stated in 2020 and has proposed four values including dignity, growth, fairness, and children first. The Beijing principle suggests that the development of AI should protect and promote the physical and mental safety of children, protect them from physical and mental violence, injury or abuse, neglect or negligent treatment, maltreatment, or exploitation. And responsible AI should help combat child trafficking, indecency, and other crimes. The collection of information on children should ensure their guardians’ informed consent and avoid illegal collection and abuse of children’s information. Responsible AI systems should ensure that children and their legal guardians, or other caregivers have the rights to consent, refuse, erase data, and revoke authorizations (BAAI, 2020).

Clarke (2019) proposed ten principles and claimed that these principles can be used as a basis for particular AI artefacts, systems, and applications in diverse AI industries. Microsoft has launched the Office of Responsible AI (ORA) and the AI, Ethics, and Effects in Engineering and Research (Aether) Committee to put responsible AI principles into practice (Microsoft AI, 2020). Microsoft has claimed six responsible AI principles, including fairness, inclusiveness, reliability and safety, transparency, privacy and security, accountability (ibid.). Another twelve researchers have created five major principles of responsible AI by incorporating four core principles commonly used in bioethics (i.e., beneficence, non-maleficence, autonomy, and justice) with a new principle named explicability that combines intelligibility and accountability (Floridi et al., 2018). They believe that the four bioethical principles can adapt well to the ethical challenges of AI. The key contribution of this statement of responsible AI principle is that they involve the original intention of the creation of any technology, which should be a benefit for and no harm to mankind. Particularly for digital health, organizations should respect the principles of bioethics. Although people are passive receivers in the digital world which means they do not have the power to refuse embedded technologies, information, and interruption, organizations still need to prioritize the autonomy of people throughout the design and execution of AI. Table 1 comprehensively depicted and summarizes the three important responsible AI principles model and organize similar concepts together to help build a better understanding of the existing frameworks. This table has removed some original principles from the three groups of researchers because they were not relevant to this research and also because there might be overlapping areas with other principles, and so the table has only retained eight essential principles which were fairness, inclusiveness, reliability and safety, transparency, privacy and security, beneficence, non-maleficence, and autonomy.

**Table 1:** A summary of three important responsible AI principles model

Elements	Clarke (2019)	Microsoft AI (2020)	Floridi et al. (2018)
Justice/fairness	Process and procedural fairness and transparency should be fulfilled.	AI systems should treat all people fairly and produce fairness rather than reinforce bias and stereotype to society	Using AI to correct past mistakes such as eliminating unfair discrimination and to create shared or sharable benefits without creating new harm.
Inclusiveness	-	AI systems should intentionally engage communities.	-
Reliability and safety	Embedded quality assurance.	AI system should be consistent with designers’ ideas, organizational values, and principles. It applies to any products of the company.	-
Transparency	Ensure accountability (i.e., each entity is discoverable) for legal and moral obligations	AI systems should be understandable, and people can understand behaviors of AI, designers open to users with why and how they create the system.	The relationship between humans and this transformative technology should be readily understandable to the average person.
Privacy and security	-	AI systems should be secure and respect privacy through considering data origin and lineage, data use internal and external.	-
Beneficence	Consistency with human values and human rights.	-	The original motivation of creating AI technology is to promote the benefits or well-being of humans and the planet with dignity and sustainability.
Non-maleficence	Safeguards for human stakeholders at risk should be provided and replace that inhumane machine decision-making.	-	Be cautious against the potentially negative consequences of overusing or misusing AI technologies, for example, the prevention of infringement on personal privacy, even worse as the AI arms race. Accidental or deliberate harm should be taken seriously whether from the intent of humans or the unpredicted behavior of machines.
Autonomy	Human ceding power to machines; but all stakeholders have legal and moral obligations to assess the impacts of AI	-	A principle that the autonomy of humans to make decisions should be protected rather than delegating too much to machines.

Sources: Clarke (2019). *Principles for Responsible AI*. https://arxiv.org/abs/2101.02032. Accessed 1 November 2020

Floridi et al. (2018). AI4People-An Ethical Framework for a Good AI Society: Opportunities, Risks, Principles, and Recommendations. *Minds and Machines*, 28(4), 689–707

Microsoft AI, 2020. *Responsible AI*. https://www.microsoft.com/en-us/ai/responsible-ai?activetab=pivot1:primaryr6. Accessed 4 October 2020

### Social Media as Channels for Disseminating Digital Health Information

Three directions of AI use in social media are stated as follows, involving discovering health topics, bio-surveillance monitoring and discovering self-management information.

#### Discovering Health Topics

In disease surveillance and epidemic detection, control of chronic diseases, behaviour monitoring, and public health communication and education, health intelligence contributes to these areas (Shaban-Nejad et al., 2018). Public-health officials can leverage social media websites that produce real-time data about a daily population of advanced events to discover health issues, including expected seasonal events, such as influenza, allergies, disease outbreaks, food poisoning, or a biochemical contaminant (Dredze, 2012). For example, one research uses supervised machine learning to discover the types of health topics discussed on Twitter, and how tweets can augment existing public-health capabilities. And by examining the disease words, symptoms, and treatments with the ailment, supervised machine learning uncovers 15 ailments including headaches, influenza, insomnia, obesity, dental problems, and seasonal allergies, such as the H1N1 virus (ibid.). Besides, social media content could be a valuable source of information for predicting or comparing different data sets during different periods (Stieglitz et al., 2020).

#### Bio-surveillance Monitoring

The increasing usage and public participation of social media platforms have provided the government with a chance to get access to public opinions through social media monitoring and controlling of governmental policies for the welfare of the general public (Singh et al., 2020). Monitoring online health discussion offers valuable insights into public health conditions that are not valuable in terms of more traditional methods which depend on manual transcripts from clinicians and other healthcare workers (Brownstein et al., 2009). Global Health Monitor is one of the web-based systems for detecting and mapping infectious disease outbreaks (Doan et al., 2019). The system can analyze and classify English news stories from news feed providers for topical relevance and use geo-coding information to help public health workers to monitor the spread of diseases in a geo-temporal context. Besides, social media platforms provide users’ location information for researchers to execute bio-surveillance, for instance, to direct vaccine suppliers to the areas and populations (i.e., demographic groups) where they were most needed. Overall, social media has already become one of the most useful sources for governments to make bio-surveillance monitoring while AI holds great promise for improving the delivery of health services in resource-poor areas (Wahl et al., 2018).

#### Sharing Self-management Information

Social media data forms new public-health capabilities, particularly for those people who are reluctant to discuss their issues with healthcare workers (Dredze, 2012). For example, negative perceptions and discrimination towards persons with mental illness are substantial and widespread (McClellan et al., 2017) which hinders those persons to seek help, however, social media information provides open resources to them. Among all of the health issues, mental health disorders are affecting a substantial portion of people worldwide. More than 80 % of people globally are experiencing mental health conditions, including individuals who are experiencing neurological and substance use disorders (Ghebreyesus, 2019). Social media platforms have become a hidden place for people with mental health disorders to share and absorb associated mental health experiences, feelings, and medical suggestions.

### The Appliance of Social Media and AI for Health Care

Traditionally, public health requires collecting clinical data aggregation from clinical encounters, which are expensive, time-consuming, and slow (Dredze, 2012). However, the advances of AI capabilities in computational and data sciences assist us to extract and analyze social media data to estimate the incidence and prevalence of different health conditions, as well as related risk factors (Shaban-Nejad et al., 2018). The application of AI to participatory health informatics can encompass the use of physiological data used from text-related data, electronic health records, social media, wearable devices, and clinical trials. Current literature has done piles of empirical research on the appliance of social media and AI for improving public health (see Table 2).

**Table 2:** Select studies on combined social media and AI use on health issues

Study	Sample	Research context	Objects of analysis	Position of responsible AI principles in the conceptual model	Research gaps/Theoretical contributions	Major findings
Rocha et al. (2018)	A total of 103 responses from GenomeConnect and Simons VIP registry participants	Rare disease community and genetic testing	Two online patient registries	Privacy. Autonomy. Reliability.	There is a paucity of literature characterizing the potential for communication, networking, privacy and membership preferences, and support needs for people with rare genetic diagnoses. This preliminary work could inform the design of more robust and nuanced research of rare disease communities collectively or specific rare disease communities individually.	• There is broad variability between individuals’ privacy preferences, according to experiences, concerns, and adaptation to their diagnosis or genetic rest results. • Patients wish to have some control over the visibility of the information they share. • Genetic counselors should provide patients with guidance about reliable social media resources for information.
Denecke et al. (2019)	22 articles and 12 clinical trials involving AI in participatory health contexts	Participatory health informatics	Seven databases and online forum (clinicaltrials.gov)	Transparency. Beneficence. Privacy.	Although AI for supporting participatory health is still in its infancy, there are a number of important research priorities that should be considered for the advancement of a field such as the psychosocial wellbeing of individuals and wider acceptance of AI into the healthcare ecosystem.	• AI may require the design to be embedded deeply or even invisibly in patients’ daily routine. • The analysis of social media data with AI can provide new insights into patient health beliefs and perspectives on their health, healthcare use, and efficacy and adverse effects of drugs and treatments. •The ethical and practical privacy issues using healthcare data (such as medical images, biological data, experiential reporting, and physiological data) need to be urgently addressed by health systems, regulators, and society.
McClellan et al. (2017)	176 million tweets from 2011 to 2014 with content related to depression or suicide	Depression or suicide	Twitter activities: expected response to planned behavioral health events and unexpected response to unanticipated events	Beneficence	Although ARIMA models have been used extensively in other fields, they have not been used widely in public health. The findings indicate that the ARIMA model is valid for identifying periods of heightened activity on Twitter related to behavioural health.	• Spikes in tweet volume following a behavioral health event often last for less than 2 days. • By monitoring social media communications and timing dissemination of information about mental health, prevention and treatment initiatives can be taken by government agencies and public health organizations.
Tutubalina, and Nikolenko, (2018)	217,485 reviews from authors tagged as ‘patient’	High blood pressure, pain, depression, chronic trouble sleeping, attention deficit disorder with hyperactivity	WebMD.com, a health information services website that provides credible information, supportive communities, and in-depth material about health subject	Reliability and safety	Medical applications, demographic information regarding the authors of reviews such as age and gender is important, but existing studies usually either assume that this information is available or overlook the issue entirely. The study found that convolutional neural networks perform best in predicting demographic information and topic models provide additional information and reflect gender-specific and age-specific symptom profiles.	• While neural networks in this kind of NLP-related problems perform better in terms of the classification/regression objective, topic models learn and provide extra information that may lead to interesting observations relevant to the underlying healthcare application.
Ahmed et al. (2009)	214,784 tweets from 28 April to 29 April 2009	H1N1 pandemic	Twitter data were retrieved from the Twitter Firehose API via a licensed reseller, Visibrain (n.d.)	Reliability and safety	Novel insights were derived on how users communicate about disease outbreaks on social media platforms. The study also provided an innovative methodological contribution.	• Twitter data could be utilized by library professionals for developing a better understanding of public views on health-related topics. • Using an in-depth qualitative method such as thematic analysis when analyzing social media data may lead to greater insights. • Misunderstandings of medical advice can lead to dangerous consequences and must be understood carefully.
Sumner et al. (2019)	95,555 social media posts and articles about an alleged suicide game were collected	Suicide	Twitter, YouTube, Reddit, Tumblr, and blogs, forums, and news articles.	Beneficence, Non-maleficence	Social media messages and online games promoting suicide are a concern for parents and clinicians. The study provided a better understanding of the degree to which social media data can provide earlier public health awareness.	• Novel online risks to mental health, such as pro-suicide games or messages, can spread rapidly and globally. • Better understanding social media and Web data may allow for detection of such threats earlier than is currently possible.
Booth et al. (2018)	Monthly outpatient mental health visits	Mental health awareness and stigma reduction	The inclusion of Twitter into the 2012 Bell Let’s Talk campaign.	Non-maleficence	The study provided important methodological implications for researchers wishing to ascertain the efficacy of social media and other digitally enabled media campaigns operated at population level.	• The 2012 Bell Let’s Talk campaign was temporally associated with an increase in the rate of mental health visits among Ontarian youth.
The current study	25 social media practitioners from health industry.	Responses of interviewees regarding responsible AI	Weibo, WeChat and other health communities, social media pages of medical consultation software.	Eight principles	A systematic discussion about responsible AI principles is limited; the application of responsible AI in facilitating digital health through social media is scarce; associated practical investigations of responsible AI are absent and therefore the research lacks an empirical foundation.	

Digital technologies are used in digital health initiatives including various types of technology, including those designed for information sharing, communication, clinical decision support, ‘digital therapies’, patient and/or population monitoring and control, bioinformatics and personalized medicine, and service user health informatics (Coiera, 2015). A survey showed that the vast number of experts, more than 75 %, use Twitter data, and more than half prefer to use regression algorithms to do social media prediction, but not all forecasting models can predict accurately, and prediction appears to be reliable on the affiliated field (Rousidis et al., 2020). Although the social media population comprises only a specific fraction of the population, the reach of its posts can cover broader impacts through social multiplier effects (McClellan et al., 2017).

Several existing pieces of literature have researched health-related studies associated with AI use on social media through the following ways: simple frequency analysis, content analysis, semantic analysis, supervised learning, and a major analytic approach (i.e., time series analysis and forecasting techniques) (Briand et al., 2018; McClellan et al., 2017; D’Alfonso, 2020; Rousidis et al., 2020). The time series analysis has some limitations such as the lack of ability to recognize sarcastic or humorous tweets, but it could be refined if combines with sentiment analysis (McClellan et al., 2017). Briand et al. (2018) combined supervised learning and information retrieval methods to analyze user-generated content in social media for the early detection of mental health conditions. McClellan et al. (2017) applied the autoregressive integrated moving average (ARIMA) model to identify deviations of social media data from the predicted trend in real-time and forecast time series data. They developed a model to identify periods of heightened interest in suicide and depression topics on Twitter.

AI is being applied in mental health by natural language processing (NLP) of clinical texts and social media content. The language people use, and our vocalizations are believed to indicate our psychological states, therefore, the NLP technique enabled by AI and machine learning technologies has been applied to examine the associations between language/voice feathers and mental health (D’Alfonso, 2020). By analyzing linguistic characteristics of social media contents, researchers can produce a machine learning model that can be used to forecast an individual’s mental health earlier than traditional methods, for example, another important issue in the Digital Therapeutic Alliance (DTA), which incorporates a therapist into a patient journey when offering therapeutic interventions through a smartphone, a web page, or a sophisticated conversational agent (ibid.). Therefore, mental health professionals can tailor their efforts accordingly.

### Consumer Trust Theory

People are highly motivated to scrutinize health information online, and the credibility of this health information judgment is correlated more with information characteristics rather than personal health status (Ye, 2010). Consumers’ lack of trust during the online navigation process is manifested in many aspects, such as their concerns that the personal information would be sold to third parties beyond their knowledge or permission (Hoffman et al., 1999). Although many organizations are aware that lack of trust might lead to consumers’ unwillingness to engage in the relationship exchange on social media, they are still reluctant to ask consumers to opt-in. Because of these worries, most consumers would opt out of informed consent. And, even though such websites have told users explicitly that they are tracked and recorded, in many cases, consumers are not allowed to reject some permissions if they need to use the social media websites.

The advancement of AI capacities enables the flourish of data mining and data warehousing opportunities. Social media gather an unprecedented number of personal data that raises concerns to those consumers with profound distress or particularly, mental health crisis (Gooding, 2019). Personal transaction information such as identity, credit history, addresses, and other information such as searching history, internet sited visited, preferences, and even illness information are leveraged along with each click of consumers. Hence, for website designers, runners, and market practitioners, respecting consumers’ rights to data ownership on the Internet can be the priority in earning consumers’ trust. A comparison study among three European countries revealed that taking consumers’ perception of trust into account is important in developing, launching, and marketing health-enhancing, non-edible products (Puhakka et al., 2019). Admittedly, consumer behaviour theories have well acknowledged that consumers perceive risks in their behavioural intention. Rather, this essay will examine the role of consumer trust level in influencing the effectiveness of responsible AI principles complementation. If consumers do not trust responsible AI practices, the efforts made by the managers would be in vain. And this assumption is rooted in that even though responsible AI practices play a complementary role in determining users’ actual behaviour, they may also have a deep impact on the activities’ performance.

### Theory of Information Sharing

The information-sharing theory (IST) is based at the outset that “organizational culture and policies as well as personal factors that can influence people’s attitudes about information sharing” (Constant et al., 1994). The purpose of IST is to understand the factors that support or constrain information sharing in technologically advanced organizations (Jarvenpaa & Staples, 2000). The human attitude (i.e., willingness to share, its antecedents, and role) contributes to improving information sharing quality. According to a social-psychological study about the willingness to share information, trust, commitment and reciprocity were considered as important antecedents that influence the willingness to share information with varying effects; access to proper IT capabilities influences the willingness as does life satisfaction (Zaheer & Trkman, 2017). IST draws its roots from social exchange theory (SET) issues (e.g., trust, commitment, reciprocity, and power) and social psychological factors of the life satisfaction of individuals (i.e., attitudes, feelings, and self-identity) to examine the effects on people’s intentions to share information (Constant et al., 1994; Wu et al., 2014; Zaheer & Trkman, 2017). And the IT infrastructure capability assessable to relevant stakeholders facilitates their willingness to share quality information (e.g., timely, accurate, adequate, complete, and reliable) on such platforms (Zaheer & Trkman, 2017). Task interdependence, perceived information usefulness, the user’s computer comfort, information ownership, and the propensity to share are strongly related to the individual’s use of collaborative media (Jarvenpaa & Staples, 2000). The more interdependent a person’s work is on others, the higher the needs of self-interest and reciprocity are and therefore people are more likely to share (ibid).

There are three types of information sharing: operational, tactical, and strategic (Rai et al., 2006). Operational information sharing concerns managing the flows of materials, components, and finished goods in a way to optimize production-related activities; tactical information sharing focuses on collaborative partners to improve decision quality; strategic information sharing incorporates group members in a strategic form for gaining competitive value on the industry-wide structure.

The theory of information sharing was used in social media analysis. Social media is primarily used as a personal learning space but is also used as a knowledge management tool and to develop communities for information sharing. Privacy has been identified as the main concern for users of a personal learning space from the disclosure of potential benefits, long-term use, the variety of personal artefacts to a wide range of audiences (Razavi & Iverson, 2006). For example, according to the survey, participants confirmed that cautious feelings emerged when they join a new community and the trust level improves which leads them to share more freely after a longer period (ibid.). To create privacy management mechanisms for personal learning, spaces should be based on users’ mental model of information privacy (i.e., privacy concerns, privacy strategies, and privacy needs). Additionally, the understanding and practice of information sharing have become increasingly significant for organizations to keep competitive and facilitate profitability (Hatala & George, 2009). This paper integrates the technology acceptance model, consumer trust theory, and information sharing theory to answer the aforementioned research questions in Section 1.

### Integrating Consumer Trust Theory and Information Sharing Theory

There are many overlapping areas between consumer trust theory and information sharing theory. Trust was considered as an important antecedent of information sharing theory that influences people’s willingness to share information (Zaheer & Trkman, 2017), which is consistent with consumer trust theory that believes lack of consumer trust would hinder people’s communication in the online communities (Hoffman et al., 1999). The theory of information sharing also recognizes the impact of privacy that reflects in the consumer trust theory with the emergence of data mining and data storage that worsens the privacy protection on social media. Privacy has been regarded as the most significant factor between these two theories (Razavi & Iverson, 2006). Besides, the ultimate goal of these two theories is to help organizations to increase competitive competence, and from the consumer’s point of view, the premise of theories is to benefit humans in the digital health domain through social media. The antecedent of reciprocity can be reflected in the beneficence of responsible AI while the increase of trust positively influences people’s mental health. Therefore, integrating consumer trust theory and information sharing theory assists us in better understand how responsible AI principles impact initiatives of responsible AI from the consumer level to the organizational level.

### Technology Acceptance Model

The technology acceptance model (TAM) was introduced in 1986, revised in 1989, and has evolved as a significant model in understanding and predicting human behaviour concerning the potential acceptance or rejection of the technology (Davis, 1985; Davis, 1989; Lee et al., 2003; Rauniar et al., 2014; Marangunić & Granić, 2015; King & He, 2006). This model assumes that an individuals’ technology acceptance is determined by two major factors: perceived usefulness (PU) and perceived ease of use (PEOU) and a dependent factor behavioural intention (BI) (King & He, 2006). The intention, in turn, is determined by an individual’s attitude (A) towards the technology and perceptions concerning its usefulness (Szajna, 1996). Some researchers have examined the TAM model in the health care realm. For example, Holden and Karsh (2010) reviewed the application of TAM by analyzing over 20 studies of clinicians using health IT for patient care. They found that the relationship between PU and BI or actual use of health IT is significant in each test, which implied that to improve the use and acceptance, the health IT must be perceived as useful. Designers, buyers, and other stakeholders involved with AI are advised to use TAM to assist the design or purchasing process, training, implementation, and other activities (Holden & Karsh, 2010). Another research on social media user’s attitudes found that utilitarian orientations of PU and trustworthiness of a social media site are crucial to use intention and actual use, and user engagement on social media needed to be considered (Rauniar et al., 2014). Similarly, researchers found that encouraging a positive attitude of the technology’s usefulness toward using technology is crucial, which indicated that information sessions and training on telemedicine should concentrate on the efficiency and effectiveness of technology on improving physicians’ patient care and service delivery rather than on the steps or procedures of the actual use of the technology (Hu et al., 1999).

According to Marangunić and Granić (2015), TAM has experienced four major types of modifications in recent years, incorporating external predictors (e.g., technology anxiety, prior usage, and experience, self-efficiency, and confidence in technology), factors from other theories (e.g., expectations, user participation, risk, and trust), contextual factors (e.g., cultural diversity and technology characteristics) and usage measures (e.g., attitudes towards technology, usage perception and actual usage of technology). Users’ anxiety, felt risks, trust crisis, technology characteristics and their attitudes towards AI, in this case, might be valuable considerations when users are assessing the responsibilities of AI in social media. People felt anxious about the emergent technologies particularly when the technology interferes with their life and there might be some uncertain risks such as the unconsciously exploited human rights and invasion of privacy. Users are less likely to trust the technology and pertinent changes. AI technology itself in social media is intangible and users cannot see, touch, and feel it, but they can experience the changes that occurred. Consequently, TAM theory is of great importance in analyzing the research question for this research. The authors extended the examination of the issue through comprehensive consideration of the TAM and its associated modifications to assess the performance of social media marketing.

### Ethical Use of Data

The data is the core in the information era and the use of the majority of advanced technologies including AI. Burkhardt et al. (2019) clarified that when AI has been quickly emerging as a new tool for CEOs to drive revenues and profitability, CEOs should offer guidance to enable analytics teams to develop and use AI in an ethical way. Data acquisition should be aligned with stakeholders’ expectations for the use of their data; dataset should reflect real-world populations rather than excluded data from minority groups; fairness should be considered in the development process such as data selection, feature selection, and model construction and monitoring; AI teams should use the simplest performance model and latest explicability techniques to meet different groups’ demands. Ethical use of data is closely intertwined with responsible AI principles both of which are commonly accepted guidance.

Ethical use of data, however, is seemingly superficial in actual applications of advanced technologies and requires fortified inspection. Cheng et al. (2021) recognized data dignity as an urgent issue that helps users to identify the benefits and risks concerning their digital presence and personal data, informs users of why and how their data will be used and allows users to negotiate the terms of using their data. Ethical data becomes a key issue for the responsible development of AI in social media and is particularly significant for health industries. Each stage of collecting and processing AI data must be ethical. Data collectors and processors should deliberate on the responsible AI principles, including fairness, inclusiveness, liability and safety, transparency, privacy issues, beneficence, non-maleficence, and autonomy.

## Methodology

This paper performed a discovery-oriented research instrument, qualitative interviews to construct study-specific sets of questions that are open-ended in nature so the participants can contribute their insiders’ perspectives with little or no limitations imposed by more closed-ended questions (Chenail, 2011). Before the interview started, this paper formulates the purpose of this investigation and the conception of the theme to be investigated, obtain a pre-knowledge of the subject matter, plan the design of the study, and conduct the interviews with a reflective approach to the knowledge sought and the interpersonal relationship of the interview situation (Kvale, 2007). The participants selected in this research all have some working experience in sharing information on social media in China. They either promoted the medical service or products on social media platforms such as Weibo, WeChat, and relevant health communities or answered patients’ enquiries, monitoring public health on social media pages of professional medical consultation software. AI technology was deeply embedded in social media and people working for them are enabled by AI capabilities to improve their working performance.

### Epistemological Approaches to Responsible AI

Qualitative analysis in academic marketing involves approaches that match across the centre of the spectrum, extending to the construction end (Hanson & Grimmer, 2007). The purpose of such research is to develop insights rather than measure, to explore rather than pin-down (ibid.). Interviews allow for a comprehensive investigation of human activities that can be aimed at practical, complex, and commercially important issues such as consumer preferences (Gummesson, 2005). Due to the characteristics of this research, the authors draw upon the interpretive methodologies which aim to achieve substantive meaning and understanding of how and why questions regarding the phenomena under investigation in the marketing, in managerial and consumer contexts (Carson et al., 2001). To gain validity and trustworthiness in this qualitative research, the authors pay close attention to the careful use, interpretation, examination, and assessment of appropriate literature; careful justification of the qualitative research methodologies employed in this research, and specifically the appropriateness, merits, and values; careful structuring of interview analysis to ensure comprehensive and descriptive evaluation and assessment (ibid.).

### Sample Design

In-depth interviews were conducted online among participants from Chinese health industries, including one hospital, one healthcare centre, and one medical centre, to collect primary data that reflect 25 social media executives’ and general staff’s insights, working experience, and concerns towards responsible AI. (see Table 3) Clinicians from a Chinese hospital were included because they were dedicated to communicating with patients through social media pages and answering enquiries from them as part of their job duties. Social media executives and staff from a healthcare centre and a medical company were interviewed because they were broadcasting their service and product information on social media, as well as monitoring and looking after the health situation of their users frequently. Depth interviews focused on social media activities relating to health information searching and generating, the user experience of platforms, as well as their recognition of responsible AI efforts. Depth interviews of one to two hours’ length were conducted with interviewees who were identified using theoretical sampling through peer introductions and snowballing techniques. They have provided a wealth of professional insights and anecdotal evidence supporting the face validity of our propositions.

**Table 3:** Profile of 25 participants in the interviews

Participants	Number of participants	Occupations of participants	Industry sector
Participant A-G	7	Social media staff (general)-Clinician	Hospital
Participant H-I	2	Social media executives	Hospital
Participant J-M	4	Social media executives	Healthcare center
Participant N-S	6	Social media staff (general)- Clinician	Healthcare center
Participant T-U	2	Social media executives	Medical corporation
Participant V-Y	4	Social media staff (general)	Medical corporation

### Proposition Development

The set of propositions shown in Table 4 connects the eight responsible AI principles identified by Microsoft AI (2020) and Floridi et al. (2018) to the social media platforms for the use of digital health (Dredze, 2012). The research propositions are informed by three sources: current research on responsible AI principles and practices; current health research on social media; and depth interviews with 25 social media practitioners that have related working experience of social media communities. The authors integrated three overlapped principles (i.e., justice, explicability, accountability) and remained eight major principles (i.e., fairness, inclusiveness, reliability and safety, transparency, privacy and security, beneficence, non-maleficence, and autonomy). Companies have endeavoured to translate these principles into actionable practice and sometimes fall short of dictating specific actions in practice, so a variety of solutions are required (Peters et al., 2020). Qualitative studies in this paper are carried out among social media marketing executives and staff in healthcare industries to examine the execution of responsible AI principles.

**Table 4:** Propositions of the organizational implications for responsible AI

No.	Responsible AI principles	Social media channels for digital health information	Organizational propositions	Moderator
1	Fairness	Discovering health topics. Bio-surveillance monitoring. Sharing self-management information.	P1: The principle of fairness facilitates the performance of social media marketing	Consumer trust. Data quality. P9: Trust and data quality moderate the performance of social media marketing
2	Inclusiveness		P2: The principle of inclusiveness facilitates the performance of social media marketing	
3	Reliability and safety		P3: The principle of reliability and safety facilitates the performance of social media marketing	
4	Transparency		P4: The principle of transparency facilitates the performance of social media marketing	
5	Privacy and security		P5: The principle of privacy and security facilitates the performance of social media marketing	
6	Beneficence		P6: The principle of beneficence facilitates the performance of social media marketing	
7	Non-maleficence		P7: The principle of non-maleficence facilitates the performance of social media marketing	
8	Autonomy		P8: The principle of autonomy facilitates the performance of social media marketing	

Sources: Peters et al., 2020; Sanches et al., 2019; Olson, 2018; D’Alfonso, 2020; Floridi, 2018; Morley et al., 2020; Shaban-Nejad et al., 2018; Brownstein et al., 2009; Dredze, 2012; Hoffman et al., 1999

### Construction of Interview Schedule

We constructed the interview items from the abovementioned conceptual categories about the responsible AI principles and social media. These items determined the main structure stems formulated for our interview schedule (see Table 5). Our discussion is organized by responsible AI principles and starts with developing propositions related to social media platforms for digital health use. This paper also proceeded to discuss the moderating role of trust and data quality, and ascertain the validity (i.e., whether an interview study investigated what is intended to be investigated), reliability (i.e., how consistent the results are), and generalizability (i.e., how to generalize the findings of an interview study to larger groups) of the interview findings (Kvale, 2007).

**Table 5:** Construction of Interview Schedule for Domain of Responsible AI principles to social media application

Categories of responsible AI principles	Item no.	Scheduled question stem and probe	Reference for category development
Fairness	1	Can you share with me the act of fairness regarding the use of AI and social media in your organization? Probe: How is the effectiveness?	Peters et al., 2020; D’Alfonso, 2020.
Inclusiveness	2	What have your organization done for engaging social media users? Probe: How did they react?	Sanches et al., 2019; Osatuyi, 2013
Reliability and safety	3	How to ensure the reliability and safety of AI in your organization? Probe: Did it work?	Olson, 2018; D’Alfonso, 2020.
Transparency	4	What have your organization done for improving the openness of AI? Probe: How did users react?	Olson, 2018; Peters et al., 2020; D’Alfonso, 2020.
Privacy and security	5	How to ensure the privacy and security of AI in your organization? Probe: Did it work?	D’Alfonso, 2020; Dredze, 2012; Razavi & Iverson, 2006.
Beneficence	6	Do you think the AI in your organization has benefited the users? Probe: How to balance the interests between organization and users?	Floridi, 2018; Clarke, 2019; Razavi & Iverson, 2006.
Non-maleficence	7	How to ensure the non-maleficence of AI in your organization? Probe: Did it work?	Floridi, 2018; D’Alfonso, 2020
Autonomy	8	How to ensure the autonomy of AI in your organization? Probe: Did it work?	Morley et al., 2019

## Analysis of Data and Discussion

### Analysis of Social Media Marketing Towards Fairness

AI systems should be responsibly designed, developed, and deployed with appropriate safeguards such as procedural fairness (Lyons et al., 2021), and fair AI should not lead to discriminatory influences on humans associated with race, ethnic origin, religion, gender, sexual orientation, disability or other situations (Benjamins et al., 2019). Fairness is a complex ethical principle that is closely related to justice and equality, though they are quite the same as either (Peters et al., 2020). This concept captures the fair distribution of benefits, risks, and costs to all people irrespectively of social class, race, gender, or other forms of discrimination (Sanches et al., 2019). In terms of protected targets, the fairness matrix is divided into three categories, including individual fairness which means each person is treated equally, and group fairness which implies that different groups such as women and men are treated equally, as well as subgroup fairness (Cheng et al., 2021). The principle of fairness may contribute to affirmative action and extra support for a group (Peters et al., 2020). However, the research on whether the fairness of AI technology use on a variety of social media platforms remains limited. The authors have interviewed social media practitioners and found some divergent insights into fairness.

Personalized notifications on our social networking sites will be based on AI’s learning from their psychology and behavioral patterns, as well as AI’s understanding of each audience. Consumers don’t feel unfair when using social media but may feel extremely targeted. (Participant D)

Based on background information, keywords searched, browsing records and pages, social media platforms sent processed, analyzed, and customized health messages, such as health ads or other health bloggers to audiences. A certain degree of prejudice and stereotypes therefore emerges, but the degree may be reluctant from time to time, one user to another, for example, the machine could infer that a single girl was pregnant depending on the already-known age information. People may think that data do not suffer from human bias itself, but the truth is that all decisions are deeply impacted by society and stereotypes (Benjamins et al., 2019). Hence, marketers have acquainted that consumers may feel annoyed and offended by the excessively targeted experience.

Our platform would introduce products mistakenly sometimes, just according to age and gender of consumers, therefore, for example, a single girl has received messages of pregnancy test products because of AI’s misunderstanding. (Participant M)

Companies have created complicated and intelligent hierarchical membership systems to earn the interests of different categories of members through subsections. They offer completely different consumers different products, services, and information. The dark side of these customized offers might be injustice, discrimination, and harm. The marketing strategy of targeting does not involve unfair intention, but the targeting strategy and relevant AI efforts by nature segment audiences into diverse classifications, which makes audiences being treated differently and unfairly. AI has its systems of execution standards created by AI designers.

We have a big data business, such as a user pricing system, there will be some “price killers” (e.g., three people may search for different prices in the same place at the same time for a product). To some extent, these policies might be against the principle of fairness. (Participant R)

These user’s hierarchy systems, such as drug price system and membership system in the online communities where there will be some “price killer policy” (e.g., three people have searched for different drug prices in the same place simultaneously). These policies are against the principle of fairness. AI learned so much information from mobile phones through machine learning, then marked prices for each piece of information and sent different notifications to grouped audiences. Organizations intend to strike a balance between the principle of fairness and the major objectives of profits.

Overall, companies are more likely to promise fairness, but the essence of companies is for-profit which cannot be neglected. The value that companies want might not be consistent with what consumers pursue, and therefore many consumers remain doubtful towards companies’ promise for fairness, which becomes a tough question for organizers. There might be some stereotypes towards technology and companies. With the unified technique of fairness, the process of evaluation and correction for fair AI would be easier (Benjamins et al., 2019). A company was assumed to wear a cap of ‘fairness’, but it tends to be difficult to define whether the judgment and activities are fair or not. Companies use AI to make a profit by intelligent grading. According to some interviewees, they believe that AI is a complete tool and means for companies to make profits. Many behaviours of companies, including AI efforts, are more likely to be what companies fabricate for greater profits. The extreme pursuit for fairness is more likely to damage the interests of the company and negatively influence the effectiveness of social media marketing. Hence, this finding is partly contradictory to the principle of fairness. But still, fairness should be an important principle for companies to follow.

- Proposition 1: The principle of fairness facilitates the performance of social media marketing

### Analysis of Social Media Marketing Towards Inclusiveness

Inclusiveness means engaging poor consumers and producers in the development, production, and use of the AI ecosystem, and helping them benefit from such technology. Inclusiveness can be achieved through active participation in the design and innovation process, increased access of the poor to technological services, and therefore an increase in the self-reliance of the rural poor (Sanches et al., 2019). AI-powered companies tend to involve and engage a wide range of consumers in depth through technological advantages and innovation. Respecting the characteristics of audiences with various background can be priorities regarding this principle.

Inclusiveness requires us to promote products and services based on the interests and standards of different groups of people, rather than recommending high price drugs and treatments to low-income groups, which increases our costs. (Participant L)

Social media platforms usually require users to agree to some privacy or authorization agreement before using them. These agreements also become obstacles for people to be included in the platforms particularly some privacy or authorization policies that do not fit in with users’ interests and preferences.

If they do not need the app, they would reject the license agreement which set a huge negative impact on social media marketing. Although users are entitled the rights to refuse, if they refuse, they cannot use the social media application. (Participant A)

This is a very common and contradictory phenomenon. Additionally, consumers’ concerns about the profit-oriented essence of companies prevent consumers to raise trust in beneficial technology. Overall, embracing inclusiveness on social media provides more chances for companies to engage more potential platforms and AI technology users. Data analysts and managers can pay much more attention to the features of information (such as topic, embedded video, embedded audio, and response count) shared on social media which indicates the credibility of information, to better understand how to engage consumers (Osatuyi, 2013). Besides, companies might need to improve the efficiency of technology and avoid the deviation level of costs when engaging more consumers.

- Proposition 2; The principle of inclusiveness facilitates the performance of social media marketing.

### Analysis of Social Media Marketing Towards Reliability and Safety

Doctors have been worried about the way that AI is being developed. Taking artificially intelligent bots Babylon Health as an example, it cannot conduct a relatively rigorous overhaul of the development process and some of the clinical advice was not vetted, then its effectiveness was exaggerated (Olson, 2018). The increase in perceived reliability and safety, and therefore trust in the social media platforms backed by AI technology may close the reinforcing loop as it triggers people to shift from their current health-seeking channels more to the use of responsible AI-powered platforms. Reliability and safety are intrinsically significant particularly when introducing AI to users. Responsible AI requires designers and engineers to consider that AI is for human beings, therefore AI should abide by the ethics and interests of specific groups, not harming the interests of others. In a nutshell, AI companies should be as risk-free or as controllable as possible. If AI technology is allowed to develop freely beyond the control of human beings, the problem will become extremely complicated and troublesome.

We have realized the concerns of reliability and safety from users because the function of machine learning-enabled social media platforms to remember users’ search records and accordingly predict their preferences and also show relevant results occasionally across different platforms which lead to some of the users feel unreliable and unsafe. For instance, once they searched for a health-related message on one platform, the other platform would immediately jump back to the relevant link or products, which made them felt much terrible. (Participant G)

Particularly when they searched for an embarrassing ailment and some awkward messages would continuously emerge on diverse websites and applications in a certain period albeit companies acclaim that the tracking and predicting obligation of AI is to offer much useful information. Many mobile and computer users tend to use a lot of remembering password functions on their mobile phones and browser. However, AI’s machine learning capability is so strong that makes consumers feel not much certain about whether their password and information are secure enough and not being sold to others. Many fraudulent calls derive from such unreliability and unsafety.

Our platform will let users tick boxes including privacy agreements and licensing agreements, but to be honest, not too many users would carefully look into details of these agreements, unless they are worrying, or only when it may endanger their interests, or cause extra costs. (Participant O)

In relatively fewer situations, social media users would pay careful attention to these agreements. In most cases, they are more likely to ignore these requirements, which in turn exerts pressure on platforms to improve the reliability and safety of social media platforms and AI-related technology. However, social media users are not innocent of the drawbacks of these advanced technologies. Some of them use some plug-ins to block ads on their Google Chrome and mobile devices, so that so-called unsafe information may be blocked. But it does not mean the platform and technology become comprehensively safe. In a nutshell, the principle of reliability and safety are more likely to facilitate the performance of social media marketing by increasing consumers’ recognition of technology.

- Proposition 3: The principle of reliability and safety facilitates the performance of social media marketing.

### Analysis of Social Media Marketing Towards Transparency

The transparency principle of responsible AI requires designers of AI systems to be open to users for example letting them know how the design process emerging and evolving. AI systems should be clear to users, and fully consider the user profile to accommodate them to the transparency level needed, particularly when using third-party AI technology and more relevant for applications such as medical diagnosis (Benjamins et al., 2019). When seeking a transparent algorithm, an understandable explanation of how AI operates is developed (Cheng et al., 2021). This may not only build trust from consumers but also increase the quality of relevant information generated. Building AI for healthcare requires a further reconsideration of how a specific technology is designed. These rules should involve clinicians who can type a range of probabilities of symptoms into the systems (Olson, 2018).

Giving a brief introduction to AI might be needed for some people, but if companies want to introduce the operation principle or mechanism behind AI, they need to be cautious when reaching a high level of secret or have already been involved in the level of other stakeholders’ interests. Proper introductions might be appropriate, but it needs to be classified based on the level of information.

Some can be introduced, but some had better be retained. For example, if the intellectual property rights and core technologies that are related to interests or secrets, especially regionally and nationally, are presented publicly, they will have an extremely negative impact.

Our designer could tell consumers how the principle is but can’t tell them the specific algorithm logic and process, which is a huge project that is impossible to uncover. (Participant B)

After all, AI is a machine that helps to learn people’s thoughts and behaviour which are authentic and essential information. The users of AI have certain rights to know.

We are worried about the possibilities of whether AI can be interpreted sufficiently up to the current development level of AI technology. Provided that AI is sufficiently open to the public, whether audiences can understand AI is still uncertain owing to the different levels of cognitive competence among audiences. It is more likely that users may never think of getting to know the design and operation of AI. (Participant Y)

Moreover, consumers and AI owners, as well as designers, may be standing in two completely different parties, representing different positions, which implies that the companies’ position can be quite different from consumers. There can be conflicts of interest and incorporating some places that can’t be understood either. Overall, a certain level of transparency of AI-enabled social media could close the distance between inanimate technology and its users which may have a positive influence on the performance of social media marketing.

- Proposition 4: The principle of transparency facilitates the performance of social media marketing.

### Analysis of Social Media Marketing Towards Privacy and Security

The current stage in the information life cycle, the nature of trust between the owner and the receiver of information, and the dynamics of the group or community would play a strong role in the user’s sharing attitude (Razavi & Iverson, 2006). Social media platforms have multiple specific policies for protecting consumers’ privacy and security. Firstly, on some platforms, users can choose whether to disclose their personal information. Secondly, companies or third-party service providers may use cookies and some tracking technologies (i.e., pixels, beacons, mobile application identifiers, and Adobe Flash technology) to recognize users, improve their experience, increase security, serve to advertise, and measure the use and effectiveness of services. Additionally, consumer’s navigation on the platforms could be tracked and being targeted by certain third-party advertising companies. For example, patient users might be integrated into practice management systems, making a referral, sending a prescription to a pharmacy, or sending a test to a clinical laboratory, and authorize platforms or third parties to run statistic research on individual or aggregate trends.

Consumers do have the right to opt-out of tracking policies, but the reality is that the majority of consumers might have ignored the rights and followed the systematic setting by default. Even though geographically localized information may lead to many privacy concerns, some users might not be fully informed that they might be served with location-enabled service. (Participant I)

Whether a company can establish good credibility and corporate reputation greatly depends on how well it can keep the privacy of customers. In developed countries, the probability of being pushed related searching records is relatively small, which situation is much better in developing countries. In other words, consumer privacy protection systems in developing countries are even worse. Many social media users alleged that their privacy must not be well protected. In the information and digital situation, it is very difficult to completely protect privacy unless people do not use any electronic products. And unless people do not use computers and mobile phones, as well as any other electronic devices, people can do protect our privacy. Many companies like Apple would have greatly leveraged information security as a selling point, especially after their system has been upgraded or when a new design for improving security developed. This fact shows that there must be a severe problem with the security of information.

- Proposition 5: The principle of privacy and security facilitates the performance of social media marketing.

### Analysis of Social Media Marketing Towards Beneficence

The principle of creating AI technology that is beneficial to humanity has been expressed in different ways, ranging from prospering for mankind and the preservation of a good environment for future generations (Floridi, 2018). Beneficial AI should be human-centric and at the service of society, as well as produce tangible benefits for people (Benjamins et al., 2019). For a specific social media and health sphere, the principle of beneficence might be bringing more possibilities, opportunities, alternatives for the current and potential consumers’ health. The application of AI technology in the medical field has indeed brought a lot of benefits, for example, some minimally invasive surgery with a micro camera can detect and photograph feedback for treatment. Another example is to predict what kind of disease a person will have through gene sequencing, and then carry out some early prevention.

Those people with mental disorders usually experience widespread human rights violations, discrimination, and stigma as well as overwhelming economic, social, and treatment costs, but social media along with AI access provides them with alternative treatment suggestions. Our efforts do contribute to solving some problems in society. (Participant J)

However, AI technology is a computational tool, and whether it can take effects would highly depend on the original intention of organizers. Despite these efforts that social media and AI practitioners have made so far, social media users still hold doubts and concerns towards the beneficence objective of AI technology. Some businesses of non-profit online services, which aim to collect data and use AI for public welfare, eventually make these data are in the hands of some private companies because many non-profit organizations outsource a lot of business to private companies. The benefits of AI have not been admitted widely, which might owe to the whole economic and societal development of the society that propels the visibly beneficial AI. Still many globally social problems, such as the gap between the rich and the poor, have always existed and are not being relieved much, because many poor areas still have no access to the Internet.

So far what AI has contributed seems limited, but AI can actually have more advantages than disadvantages when used properly. (Participant N)

AI can become a promoter, not a chaos creator. AI is not created for the benefit of mankind and therefore its usage depends on those who have the right to operate and use it. What companies need is to maximize the organizational interests, not to maximize the effectiveness of AI without considering the sake of companies. The interviewees commonly admitted the restricted contributions that AI had on humans, but they were not sure that the emergence and development of AI will necessarily promote the development of human society. AI should be executed under the principle of beneficence, although it is very difficult to recognize the accurate influence it has on human beings, still, it might attract much investment into this industry to broaden the beneficence of AI and social media. Perceived benefits of real-time information sharing lead to overall perceived purchase and repurchase behaviour mediated by customer orientation (Ghouri and Mani, 2019).

- Proposition 6: The principle of beneficence facilitates the performance of social media marketing*.*

### Analysis of Social Media Marketing Towards Non-maleficence

Non-maleficence refers that the creation, design, and application of AI should be cautions against various negative consequences of overusing or misusing AI technology (Floridi, 2018). AI systems should in no way lead to negative effects on human rights (Benjamins et al., 2019). Along with the promising future of AI, various risks and related warnings arise from their technological innovations, such as the recursive self-improvement of AI and the threats of an AI arms race. (ibid.) Thanks to the wide use of AI in the mobile internet industry, which makes people feel that everything is faster and more convenient in today’s life, the sense of boundary has become more unclear than before so the balance between life and work as well as social life has been broken.

Some of our users stated that AI in social media has been overused. They complained about the decreasing time for rest and less private space since they need to be ready for work and socialize anytime and anywhere. (Participant C)

The overuse of AI has made people living in overwhelming information conditions. People complain about the overuse of AI because they have received overloaded messages every day and most of them are useless and even annoying and upset. Hence, the opposite of convenience and fast is that, to some extent, AI harms people’s mental health and balanced life. In a nutshell, there is still a way for organizations to relieve the negative effects led by fast-paced life, and simultaneously to make everything more convenient, faster, and efficient.

We recognized that AI has been misused to some extent. Its original goal was to benefit mankind and promote human development, rather than to do AI for the benefits of the power and money of those who have acquired it. But the truth is that AI has been used for the sake of companies regardless of the obstructive effects it may have on people. (Participant D)

For example, many social network software, such as WeChat pay, Alipay, and bank card binding function, will form a lot of big data reservoirs to help analyze the user’s information. These platforms intend to build a huge network for you to analyze and control. In principle, these behaviours should bring as much convenience to your life as possible, but because its system is getting more complicated and large, people’s lives are becoming more inconvenient. To improve the non-maleficence of AI is more likely to facilitate the performance of social media marketing unless companies pay much attention to the natural needs of humans such as much quiet rest time.

- Proposition 7: The principle of non-maleficence facilitates the performance of social media marketing.

### Analysis of Social Media Marketing Towards Autonomy

AI must protect and enhance users’ autonomy and abilities to make decisions and choose between alternatives. Users should also be able to make informed autonomous decisions regarding AI systems. If not, users may feel that their decisions are being curtailed by the systems that they do not understand, and it is very unlikely that these systems will satisfy social acceptability, regardless of social preference that should be the goal of a truly ethically designed AI (Morley et al., 2020). A trustworthy AI can be more socially acceptable (Taylor et al., 2018).

We admitted that AI has deprived some of the users’ rights for general decisions in daily life. Users may hope to keep more of their rights. (Participant M)

Additionally, AI technology has relative stereotypes about each audience. It can help them to solve problems, but these are all based on its restricted machine learning and understanding of the audience. However, human beings are much more subjective and emotional, so there are many aspects that AI can’t sufficiently understand. So far AI is not so accurate, so social media users would have to take some risks to follow its suggestions.”

Some people have different requirements towards autonomy, ranging from diverse age generations, educational backgrounds, and cultural differences. For the elderly and the children, who have limited cognitive abilities and recognizing true from false, is where AI should entitle them more autonomy to help make decisions, rather than lead them in the wrong direction or go astray. But for young and middle-aged people, the proportion of AI interruption in the consumer decision process can be smaller. This is hard for machines based on the existing technology. (Participant F)

In a nutshell, a different group of users and events require to be allocated to determine the degree of AI participation, while AI is intelligent, it should make different efforts for different people. Furthermore, for two adults of the same age, they may need a different level of AI interruption preference. For some people, AI are required to entitle more rights and choices to help users make choices; for another user, they may desire much dominant power and only need basic services or suggestions from AI. Organizations claimed another issue is that the range of permissions is difficult to define and may need to be evaluated by specific design theories and systems. For example, providing three to four suggestions may be considered to be highly autonomous, but more than a dozen suggestions are not that autonomous.

To ensure the autonomy of social media users requires companies to segment people into different groups which might have conflicts with the excessive targeted concerns of people. Unless this issue has been addressed, the proper level of autonomy can both facilitate the performance of social media marketing and ensure recognition of the company. More importantly, humans must be empowered with greater rights to monitor and intervene in AI if necessary. This consideration derives from predictions of AI as superintelligence in the future.

Proposition 8: The principle of autonomy facilitates the performance of social media marketing.

### Consumer Trust and Data Quality as Moderators

Throughout the interviews with 25 social media practitioners, this paper concluded two important moderators they have mentioned as key drivers for the principles of responsible AI. They claimed that AI should build trustworthiness and quality of data. The basic premise that consumers can admit AI is that they trust it (Ye, 2010). Particularly when AI is helping people screen information and make decisions, the quality of the information can help users identify whether to accept AI’s suggestions or not. For example, if a consumer has searched for an anorectal disease, all of his/her search engines and social platforms will pop up all kinds of related advertisements, many of which are useless. This negative response hindered consumer’s level of use and trust towards AI on social media. So how to make AI technology more quickly and accurately find the most suitable page for him, or the solutions, is quite significant for consumers to build trust and reliability towards AI.

We know that AI should recommend information accurately and objectively. (Participant K)

However, a small group of people felt panicked when a dispensable platform sent accurate messages to them frequently, because it implied that AI understood them exceptionally well and beyond the restrictions of norms. Hence, it tends to be critically essential when companies leverage AI to communicate with consumers without disturbing and offending them. To build long-lasting trust between AI and human beings, the key is to address AI’s indifferent behaviour (Cheng et al., 2021).

- Proposition 9: Consumer trust and data quality moderate the performance of social media marketing.

**Fig. 1:**
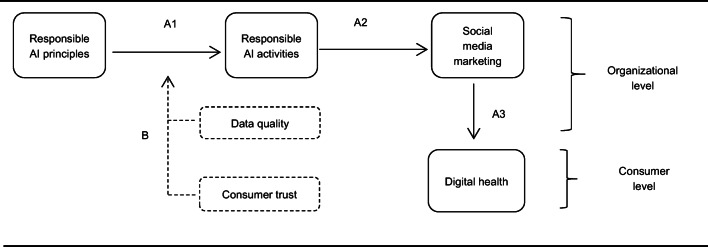
A conceptual model of the responsible AI effects. *A1: Responsible AI principles guide responsible AI activities about how to plan, design, and implement AI more responsibly. A2: Responsible AI activities are tightly integrated with social media marketing, which aims to address the ethical issues confronted with these activities. A3: The responsible AI appliance in social media contributes to public digital health, which resulted in the individual’s improvement in health conditions. B1: The level of data quality and consumer trust moderate the implementation of responsible AI principles in practices.

The above analysis and discussion on each principle and activities of responsible AI led to the development of the following conceptual model (see Fig. 1) which demonstrated the research questions and relevant factors that influence social media marketing and digital health from both the organizational level and consumer level. According to Cheng et al. (2021), three concrete objectives of trust are fairness, transparency, and safety. Data quality is what can be addressed at the organizational level while consumer trust is generated from the consumer level but rooted in the people’s acceptance of AI and influenced by many contextual factors. The interplay effects between these two moderators can help to strengthen the effectiveness of appliance of responsible AI principles.

### The Exploration of a Conceptual Model on Responsible AI

**Fig. 2:**
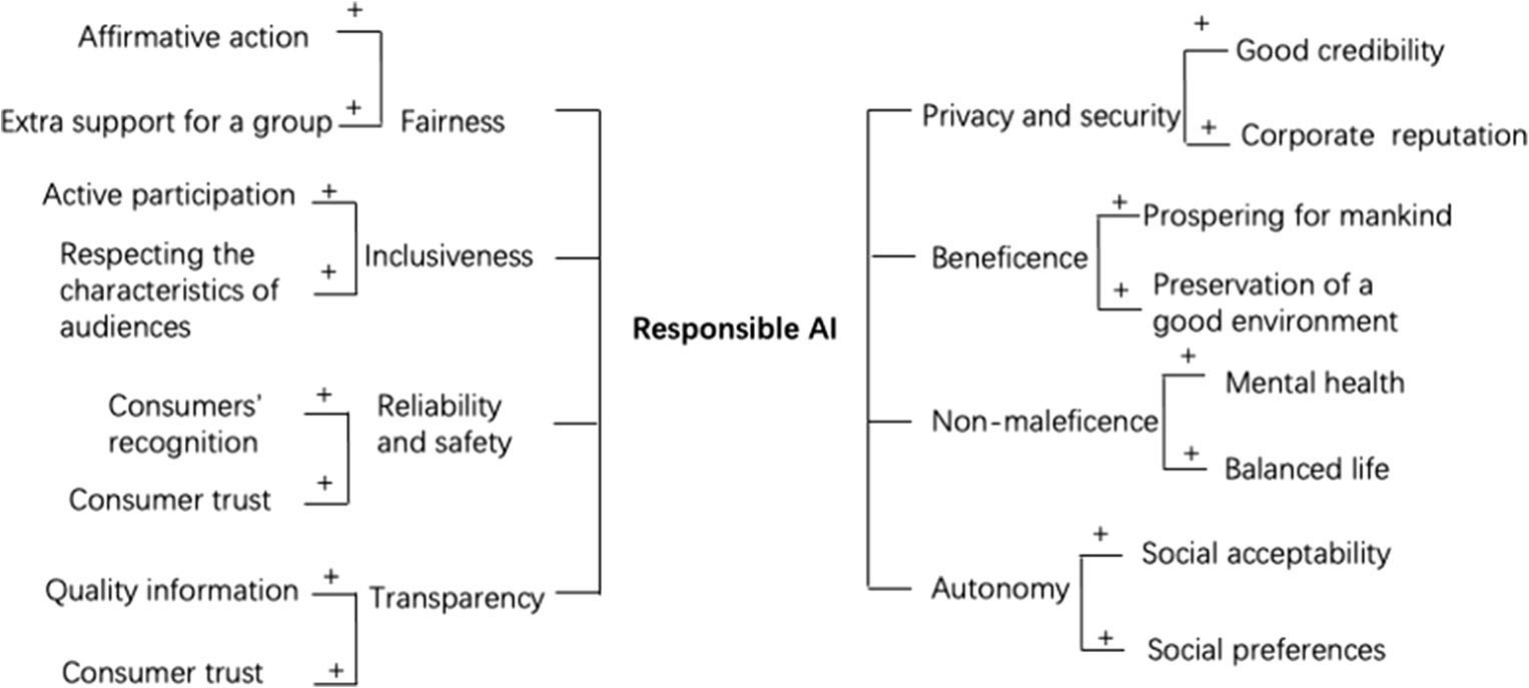
Responsible AI principles and corresponding consequences

Each principle of responsible AI and its corresponding consequences are shown in Fig. 2. It has been found in this research that the obedience of each responsible AI principle would engender significantly positive results for organizations. Firstly, achieving fairness is difficult, but organizations are required to strike a balance between profits and equity and also pay closer attention to less privileged groups. This would increase users’ willingness to engage in the technology and associated social media activities. Secondly, organizations are encouraged to involve more diverse audiences, respect their distinctive characteristics, and encourage active participation. This is in line with the born nature of responsible AI which cares about the less privileged residents and aims to help them survive and develop from the technology innovation. To broaden the potential audiences also entitles the organization’s opportunities to better understand one group and fulfill their demands. Inclusiveness requires organizations to focus on the characteristics of different groups of people, understand and respect them, which seems difficult, but these minor groups would reward the efforts. Thirdly, reliable information in social media for health care is much more important than in any other field since it is highly linked with the safety and health of each patient. Besides, data security cannot be ignored and should be listed as priorities for organizations at any time. The usefulness of technology in this research is more about the authority of the information; reliability and safety of the data are to be the most fundamental ingredients. With a higher level of reliability and safety of AI technology, users are more likely to perceive the usefulness of the technology to be willing to participate in it. Fourthly, responsible AI ought to be relatively transparent. This is not only because of the complexity of health information but also a thoughtful consideration to maintain the citizen’s rights to be informed. Users on the platforms are entitled to comprehend how their data is used, where to go, and who is to handle it to avoid any unethical or illegal use of their private information. Fifthly, the issue of protecting privacy in the digital world is more than a platitude and was regarded to be more severe than ever, as AI has leveraged some advanced skills to mimic people and even intelligently act like mankind. Seeking strict privacy protection could ensure a high level of good credibility and corporate reputation which are worthy of equity for the sustainable development of the organization. Sixthly, AI-enabled social media marketing facilitates health care for a specific group of patients, although many people are concerned about the beneficence goals of AI. Organizations can make more efforts in improving the well-being of humans and better preserving the environment. If users’ realization of the usefulness of responsible AI for health management increased, the acceptance of the technology would soon improve. Seventhly, overuse and misuse of AI are detrimental and what’s worse, the situations might happen in many timing periods of AI design and implementations which will wipe out hardly constructed user trust. Overloaded information has led to complicated and messy human life; over-processed information has worsened the invasion of privacy. The hieratical level of digital health should not be neglected like destroyed mental health and balanced life. Therefore, maintaining the non-maleficence of AI particularly when facing health issues is of much concern. Finally, the design and implementation of responsible AI should respect the subjective initiatives of humans rather than deprive them of an increasing level of autonomy. Users’ acceptability and intention to use the technology would increase by ensuring a certain level of their autonomy to choose, decide and refuse.

The results extended the responsible AI theory by forming a new set of responsible AI principles, and further validate their significances in indicating positive results for digital health practitioners when they are implementing the social media campaign. The appliance of responsible AI may greatly enhance the ease of AI use, engender a higher level of user acceptance and intention to use the technology. Hence, the TAM model also contributed to the theoretical construction of this paper. This paper leveraged the elements of the TAM model to assess the effectiveness of responsible AI use. For example, social media users would be more likely to accept the technology if it has sufficient ease of use and perceived usefulness. Additionally, a higher level of consumer trust and information quality would strengthen the consumers’ confidence in a specific technology, resulting in more user traffic and participation in the social media platforms.

## Theoretical and Practical Implications

This research proposes and empirically tests a model of social media marketing reflecting responsible AI relationships guided by specific principles, with moderating factors that are consumer trust and data accuracy. Whoever uses AI can ethically use it, since how well ethics are treated will ultimately decide how much people will embrace technology in the future. AI practitioners and researchers should be cautious about ethical issues. The more educated a criminal is, the more evil they will be. If AI becomes more and more intelligent, and people who create and use AI don’t have enough ethical knowledge, the AI-empowered world would be exceedingly troublesome. This paper has provided some theoretical and practical contributions for marketers and AI developers to consider ethical issues.

### Theoretical Implications

This paper proposed a set of eight significant responsible AI principles framework and conducted a systematic discussion. Then the study suggested the usefulness of the framework in implementing activities in social media when applied in the digital health industry. Additionally, the interviews also provide empirical evidence for the development of responsible AI principles.

In the framework, data quality and consumer trust were regarded as essential elements that moderated the principles and activities. Apart from that, this paper also contributed to the responsible AI theories and social media marketing theories from combined organizational perspectives and consumer perspectives. It implies that if an organization seeks to sustain a long-term relationship with consumers, it should abide by the eight principles and concentrate on building trust, collecting, and ethically processing the data.

The information-sharing theory is applicable in many disciplines of social science such as supply chain and information systems (Wu et al., 2014; Jarvenpaa & Staples, 2000). This paper combined consumer trust theory (Lou & Yuan 2019) to add new insights into social media and responsible AI principles to the research of the information sharing theory. The theory of information-sharing offers an understanding of the variables that enable and constrain information exchange among individuals (Zaheer & Trkman, 2017). The essence of social media platforms is important channels for people and organizations to share information. Hence, the appliance of AI technology may boost or constrain the information exchange amount and information quality which depends on the ethical issues that organizations need to obey.

Trust is the most crucial antecedent and motivation for consumers’ willingness to share (Zaheer & Trkman, 2017). The moderator in this paper, the level of consumer trust would influence an individual’s willingness to share information on social media. It is believed that investing in social values based on trust, mutuality, and respect could enable long-term organizational benefits such as corporate well-being and innovativeness (Widén-Wulff & Ginman, 2004). Good quality information, specifically reliable and accurate information sharing, is not possible on social media without trust from users (Kwon & Suh, 2005). The reciprocity consideration from information sharing theory supports the beneficence principle of responsible AI, which indicates that when people feel potential benefits of specific behaviour, they are more likely to get involved in the social media activities of organizations.

Information sharing theory requires AI developers to create user-friendly products that fit in with their acceptance and behaviour (Kim & Lee, 2006), which supports the transparency principle of responsible AI that calls for customized delivery of openness. Information sharing theory includes task interdependence as an antecedent that when people feel sharing information on social media is a social good, rather than personally costly or unpleasant, it tends to be beneficial to the organizations in the long run (Constant et al., 1994). From the theory of information sharing, power is not a significant antecedent of willingness to share; high power might engage the user to share information albeit unwillingly. For organizations, they can perceive information sharing as a loss of power so they would not be willing to share information (Zaheer & Trkman, 2017). These concerns contradicted what responsible AI wishes organizations to do. For the theory of information sharing, privacy is a key issue to remain the long-term competitiveness of organizations (Razavi & Iverson, 2006), which is consistent with responsible AI principles.

### Practical Implications

Overloaded information, false reporting, lack of signal precision, and exposure to external forces such as media interest may restrict the realization of their potential for public health practice and clinical decision-making (Brownstein et al., 2009). The current study argues that for digital health to be effective, it should not ignore the social media impact coupled with responsible AI principles by accelerating the scrutinization of individuals’ feelings, responses, and insights on social media. The results have answered the aforementioned research questions, as the results showed that interviewers are expecting AI technology and companies can prioritize consumers’ interests rather than profits, abide by ethical principles, and be in line with consumer preference and acceptance adequately when they undertake activities. The efforts that a company makes for responsible AI should also correspond to the interests of consumers and their individualized demands and situations. Our study has proposed some new comprehension of what responsible AI should like and what aspects are consumers concerned about. Companies can create different choices of AI systems to engage consumers from different backgrounds in catering to their demands; companies can construct a diverse level of openness for the AI design process and relevant knowledge for customized desires of consumers; the design and intentions of responsible AI should prioritize consumers’ interests.

The principles of fairness, inclusiveness, reliability, and safety, transparency, privacy and security, beneficence, non-maleficence as well as autonomy play an indispensable role for companies applying AI technology responsibly. Precisely, the principle of transparency and autonomy are relatively personalized options for different consumers. Reliability and safety can be enhanced by upgrading data quality and raising trust. Beneficence and non-maleficence are more likely to be rooted in the infant intentions of companies, which are extremely basic and essential. Fairness and inclusiveness tend to be related to prejudice and stereotype which may lead to consumer dissatisfaction and turndown to AI technology. What interviewees have mentioned most is privacy. The innovation of technology improved part of privacy but also eroded the level of privacy. Overall, this research offers important practical insights into ethics for AI companies and designers, which fills up with the scarce of theoretical knowledge in this field.

## Limitations and Suggestions for Future Research

Responsible AI is still in its nascent stages and there are still few commonly accepted standards (Taulli, 2021; Fosso Wamba & Queiroz, 2021). As with most empirical studies, this research is not without its limitations although every effort was made to minimize them. First, this research mostly relied on collecting data from single respondents – i.e., social media executives and general staff. Although, this paper ensured that these respondents are highly experienced employees who have years of experience working in this field and carry a wealth of knowledge, however, the fact that data was mostly collected from a single source may have had an impact on its richness as multiple participants would have provided more insights. For instance, data can be collected from various stakeholders beyond organizational employees (e.g., customers, suppliers, etc.) to provide new insights. The paper would, therefore, recommend that scholars conducting future research in this topic area focus on collecting data from multiple participants. Second, and in a similar vein, the data collected in this research was mostly gathered through qualitative, semi-structured interviews which are aligned to the exploratory nature of this research. Employing quantitative and multi-method research would not only make this research more robust but will also open new this research to new methodological directions. For example, for future research in responsible AI, this work recommends a multilevel study with clinicians and AI designers. Third, this research primarily involved examining the application of responsible AI on social media in digital health marketing. Although there is potential in our findings to be generalized to other industry sectors, there is no doubt, that the results are more applicable and useful for the healthcare sector. However, there is potential for this research to be extended to other areas of service sectors such as the use of socially responsible AI in banking and fraud, and subsequently, future researchers can also look into examining the application of socially responsible AI in other, more traditional industry sectors. Finally, there are some theoretical areas for further research based on this paper. For instance, to construct the mechanism for governance and control of privacy and build trust in social media marketing would be recommendable in the field. In summary, for the most part, the limitations of this research are those that are common to exploratory, qualitative studies. Nonetheless, given the initial stage of growth of this research area, this paper is intrigued to find out how future research in this area moves forward.
